# Linkage between Three Gorges Dam impacts and the dramatic recessions in China’s largest freshwater lake, Poyang Lake

**DOI:** 10.1038/srep18197

**Published:** 2015-12-11

**Authors:** Xuefei Mei, Zhijun Dai, Jinzhou Du, Jiyu Chen

**Affiliations:** 1 State Key Lab of Estuarine & Coastal Research, East China Normal University, Shanghai, China

## Abstract

Despite comprising a small portion of the earth’s surface, lakes are vitally important for global ecosystem cycling. However, lake systems worldwide are extremely fragile, and many are shrinking due to changing climate and anthropogenic activities. Here, we show that Poyang Lake, the largest freshwater lake in China, has experienced a dramatic and prolonged recession, which began in late September of 2003. We further demonstrate that abnormally low levels appear during October, 28 days ahead of the normal initiation of the dry season, which greatly imperiled the lake’s wetland areas and function as an ecosystem for wintering waterbirds. An increase in the river-lake water level gradient induced by the Three Gorges Dam (TGD) altered the lake balance by inducing greater discharge into the Changjiang River, which is probably responsible for the current lake shrinkage. Occasional episodes of arid climate, as well as local sand mining, will aggravate the lake recession crisis. Although impacts of TGD on the Poyang Lake recession can be overruled by episodic extreme droughts, we argue that the average contributions of precipitation variation, human activities in the Poyang Lake catchment and TGD regulation to the Poyang Lake recession can be quantified as 39.1%, 4.6% and 56.3%, respectively.

Natural lakes are large bodies of inland water, and they cover approximately 2.8% of the Earth’s non-oceanic surface^1^. Despite this small fraction of land cover, lakes are important to ecosystems at both regional and global scales^2^,^3^,^4^. However, lakes around the world are shrinking and even have disappeared in response to a warmer global climate and intense anthropogenic pressure, including the Great Lakes in the United States^5^,^6^, the Aral Sea and the Dead Sea in Asia^7^, and the Lake Chad in Africa^8^,^9^. Identifying the main drivers of lake decline is of great significance for global lake management and lake recovery, especially for the largest freshwater lake in China, Poyang Lake.

Poyang Lake basin, located in eastern China’s Jiangxi Province, has a drainage area of 162,225 km^2^ and feeds a population of up to 44 million^10^. Discharging from the south into the Changjiang River (Yangtze River), the longest river in Asia and third longest in the world, Poyang Lake annually supplies 17% of the Changjiang’s water and 2% of its sediment load, which has immense importance for the Changjiang Delta. The lake receives water from five main tributaries, the Ganjiang, Fuhe, Xinjiang, Xiushui and Raohe Rivers, and discharges to the Changjiang River at Hukou (Fig. 1, Auxiliary material, Table S1), which results in a considerable annual fluctuation of water level and creates approximately a 3000 km^2^ ephemeral wetland system^11^. These extensive wetlands provide a home for millions of migrant birds during the lake’s low water stages in late autumn and winter, including 95% of the world’s endangered white cranes (the Siberian crane, *Grus leuceogeranus*)^12^,^13^. Moreover, the variations in the lake’s output directly affect marine environments in the East China Sea.

However, since the beginning of the 21st century, Poyang Lake has experienced continuous extreme low water levels in autumn, which has led to widespread exposure and degradation of its wetlands. Following the high frequency of droughts, the submerged “thousand-eye bridge”, a 400-year-old stone bridge dating back to the Ming dynasty, has been successively revealed in recent years (Fig. 1c). Meanwhile, the earlier dry season has affected wetland vegetation growth, which has put the threatened migratory birds at risk of extinction. What is noteworthy is that the prolonged drought and exposure of wetland areas coincides with the filling of TGD (currently the largest hydroelectric dam in the world), as well as intermittent droughts and intensive sand extraction.

There is an on-going international debate about the sensitivity of Poyang Lake to climate change, TGD regulation and anthropogenic activities throughout the Poyang Lake basin. Ye *et al.* (2014) pointed out that the advance of the Poyang Lake dry season was primarily driven by climate changes in the Changjiang River basin since the 1990s and was further aggravated by TGD in the 2000s^14^. However, Feng *et al.* (2013) linked the significant shrinkage of the Poyang Lake to the impoundment of TGD, rather than precipitation or Changjiang River discharge^11^. Furtherly, Zhang *et al.* (2014) revealed that TGD induced modifications to Changjiang River flows had a much greater influence than climate variability on the seasonal dryness of the Poyang Lake^15^. Guo *et al.* (2012) indicated that under similar precipitation conditions, TGD impoundment from late July through October weakened the river forcing on the lake, which caused increase in Poyang Lake outflow and reduced lake storage^16^. Moreover, Liu *et al.* (2013) showed that the recent Poyang Lake shrinkage was a regime shift occurred in 2006, which relied on the interaction between the lake and the Changjiang River^17^. There is also an expectation that sand mining was the major determinant of recent lower lake levels in the Poyang Lake, which increased the Poyang Lake discharge ability by 1.5–2 times in low water season^18^ (Lai *et al.*, 2014). Despite several efforts to explore the causes of the Poyang Lake dryness, to date, most studies focused on the influence of TGD water regulation and climate change while few of them concerned the link between geomorphological changes around lake outlet and adjacent Poyang Lake’s recession. Furtherly, there has been no studies that attempting to distinguish and quantify the contribution of each possible factor on the recent lake recession.

Thereafter, the objectives of this study are to (1) discern the characteristics of the recession of the Poyang Lake; (2) explore the potential causes of prolonged drought and wetland exposure in the Poyang Lake basin; (3) quantify the relative contribution of each forcing on Poyang Lake shrinkage. These results are of vital value in characterizing the recession regime of the Poyang Lake and its associated wetlands, and can be applied to other large lakes in similar situations around the world.

## Results

### Recession of the Poyang Lake

**Figure 2:**
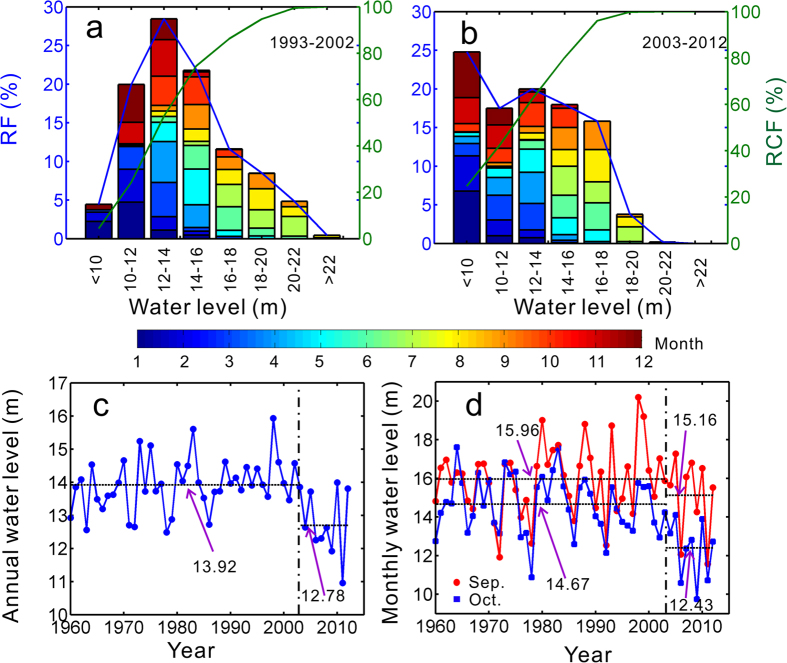
Daily, monthly and yearly water level statistics at Duchang (RF: Relative frequency; RCF: Relative cumulative frequency). The contribution of every month on each interval is indicated by the different colors in (**a–b**); (**c**) annual water level; and (**d**) September and October monthly water level. The figure was created with Matlab R2009b.

Duchang hydrological station is located in the central of the Poyang Lake, and its water level reflects the lake’s shrinkage^15^. Its daily water level over 2003–2012 is compared with levels from 1992–2002 by grouping frequency with an interval of 2 m (Fig. 2a,b). Following the filling of the TGD, the fraction of extremely low lake levels, below 10 m, increases dramatically from 4.34% to 24.79%, while the extremely high levels over 22 m have disappeared. The contributions to water level in each month change dramatically, especially in September and October. Specifically, the daily water levels in September and October range from 12–22 m and 12–18 m, respectively, in the pre-TGD period, after which they shift to 10–20 m and 10–16 m. This indicates that the dry period in the Poyang Lake began earlier and lasted longer during 2003–2012 than during 1993–2002. We note that the Poyang Lake usually enters the dry season on November 12^th^, when water levels at Xingzi drop below 12 m^19^, corresponding to 12.6 m at Duchang (Auxiliary material, Fig. S1). However, since the regulation of the TGD, the lake started to shrink in late September and approached the dry season 28 days ahead of normal, on 16^th^ October (Auxiliary material, Fig. S2).

Compared with the pre-TGD stage, the mean annual water level at Duchang decreased by 1.14 m since 2003 (Fig. 2c). Meanwhile, monthly water levels in September and October dropped by 0.8 m (from 15.96 m to 15.16 m) and 2.24 m (from 14.67 m to 12.43 m), respectively (Fig. 2d). The dramatic decrease in October water levels resulted in exposure of 1510 km^2^ of surface area (Auxiliary material, Fig. S1), which accounts for approximately 50% of Poyang Lake wetlands. In view of the significant change in hydrology in October, this study focus on the October water regime in the Poyang Lake.

### Discharge and sediment outflow from the Poyang Lake to the Changjiang River

**Figure 3:**
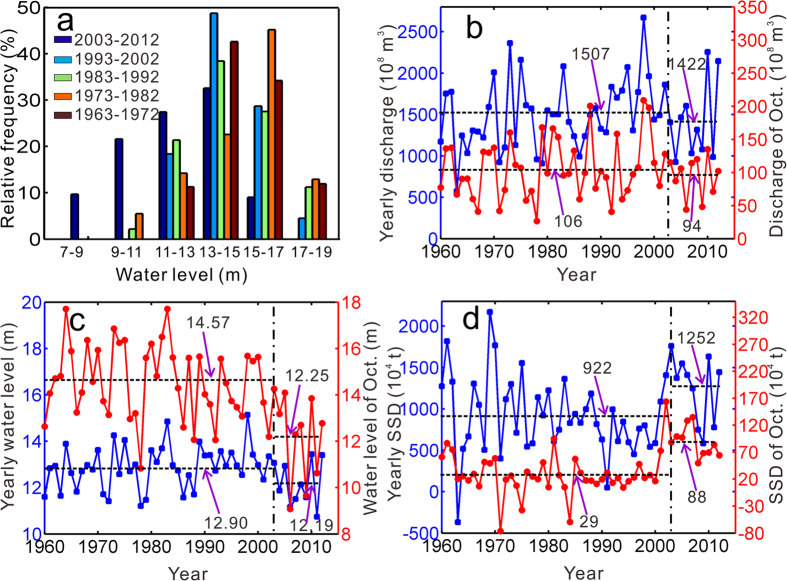
Variation in output from Poyang Lake to the Changjiang River, with (a) grouped frequency of daily water levels; (b) annual discharge; (c) annual water level; and (d) annual suspended sediment discharge. The figure was created with Matlab R2009b.

As the only outlet from the Poyang Lake to the Changjiang River, Hukou’s water level, discharge and suspended sediment discharge (SSD) directly reflect the lake-river interactions. The frequency distribution of daily water level during 1993–2012 at Hukou station follows that of Duchang, with a decrease in water level occurring since 2003 (Auxiliary material, Fig. S3). The decadal variability of daily water level distribution in October indicates a significant decrease at Hukou (Fig. 3a). Over the pre-TGD period, the daily water level mainly falls between 13–17 m in October, but shifts to 11–15 m over the last decade. Meanwhile, the distribution of extreme high water levels decreases from 17–19 m to 15–17 m.

Relative to the pre-TGD stage, the annual discharge at Hukou decreases by approximately 6%, from 1507 × 10^8^ m^3^ to 1422 × 10^8^ m^3^. Accordingly, the annual water level drops from 12.90 m to 12.19 m, suggesting a decline of 6%, which is comparable with the variation in annual discharge. However, compared with the pre-TGD period, the monthly mean discharge in October decreases by 11%, from 106 × 10^8 ^m^3^ to 94 × 10^8^ m^3^ (Fig. 3b), while the October monthly mean water level drops 16% from 14.57 m to 12.25 m in post-TGD period (Fig. 3c). Additionally, Poyang Lake supplies more SSD to the Changjiang River in the post-TGD period. It discharges 922 × 10^4^ t and 1252 × 10^4^ t SSD to the Changjiang River in the pre- and post-TGD periods, respectively, suffering a yearly loss of 330 × 10^4^ t and resulting in lake erosion (Fig. 3d).

### Geomorphologic feature of lake mouth

**Figure 4:**
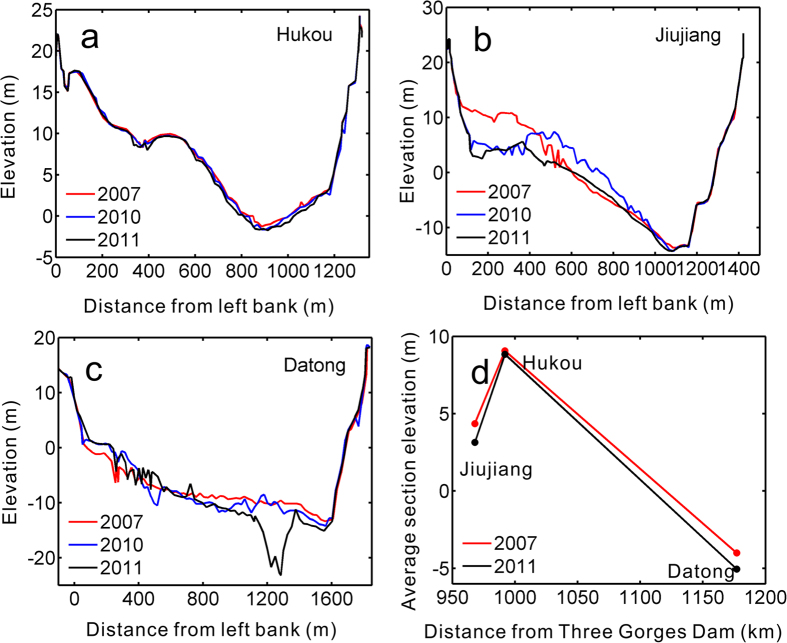
The geomorphologic evolution of transverse sections at (a) Hukou; (b) Jiujiang; (c) Datong; and (d) the variation in river-lake topographic gradients. The figure was created with Matlab R2009b.

Despite significant variations in output from the Poyang Lake to the Changjiang River since 2003, the geomorphology at Hukou station, at the lake’s outlet, only indicates slight erosion (Fig. 4a). However, the Changjiang’s riverbed (at Jiujiang and Datong) has experienced significant erosion^20^. Specifically, for the Hukou section, the largest changes appeared at an area 600 m–1200 m from the left bank, where the mean riverbed elevation decreased from 1.89 m in 2007 to 1.45 m in 2011 (Fig. 4a). For the Jiujiang section, the average riverbed elevation for the area 150 m–500 m from the left bank was 8.43 m in 2007, which drastically decreased to 3.41 m in 2011 (Fig. 4b); and for the Datong section, the most serious erosion occurred at the area 1150 m–1350 m from the left bank, which experienced an average reduction of 8.36 m in channel elevation (Fig. 4c). On average, the topographic gradient (the ratio of elevation difference to distance) between Jiujiang and Hukou consequently increased by 20%, from 0.20 × 10^−3^ to 0.24 × 10^−3^, while the topographic gradient between Hukou and Datong increased by 6%, from 0.71 × 10^−4^ to 0.75 × 10^−4^ (Fig. 4d). The obviously increased lake-river topographic gradient will greatly weaken the blocking effects of the Changjiang River and enlarge the discharge ability of the Poyang Lake.

## Discussion

Relative to its pre-TGD stage, the Poyang Lake has experienced a dramatic recession since 2003, when its water level declined by 2.24 m, from 14.67 m to 12.43 m. Poyang Lake is vulnerable to climate change and anthropogenic activities^21^. Since the beginning of the 21st century, Poyang Lake received less precipitation and tributary inflow than past years. Meanwhile, sand extraction occurred on a large scale, and the TGD, currently the largest river engineering project in the world, was implemented. Each of the above forcing has been blamed for the recent prolonged lake recession in October^11^,^15^,^16^,^17^,^18^,^22^,^23^. Here, the potential impacts of all possible factors on the lake recession are clarified and quantified.

### Impacts of climate change

Change in precipitation is the most critical factor determining the impact of climate change. Precipitation over the Poyang Lake catchment affects the lake level in two ways. Precipitation over the lake region directly affects the lake level. Precipitation over the tributaries is firstly converted to runoff and then enters the lake, altering lake level. Monthly mean precipitation over the lake region has decreased during September and October since 2003, which coincides with the recession of Poyang Lake (Auxiliary material, Fig. S4a). Relative to the pre-TGD stage, the lake gets 14.9 mm less precipitation on average than it used to in October, which accounts for 0.7% of the 2.24 m drop in lake level (Auxiliary material, Fig. S4b). Decreased precipitation from an extreme drought over Poyang Lake in 2004, accounts for a 60 mm decline in lake level, equal to 2.7% of the drop in lake level (Auxiliary material, Table S4). Therefore, precipitation over the lake region has a limited effect on lake level variations in this timeframe.

Precipitation over the Poyang Lake catchment affects the lake region in terms of flow input, 75.4% of which derive from the five main tributaries^21^,^24^. When compared to the pre-TGD period (1960-2002), the water contribution of these five sub-basins decreased by 14.2 × 10^8^ m^3^ (from 45.27 × 10^8^ m^3^ to 31.07 × 10^8^ m^3^) in October (Auxiliary material, Fig. S5), which results in an average 0.86 m drop in lake level and explains 38.4% of the lake level decline. In the case of the extreme drought in 2004 (Auxiliary material, Fig. S6), the October water discharge from all five tributaries to the Poyang Lake dropped by 23.9 × 10^8^ m^3^ compared to the pre-TGD stage, which results in 1.87 m of lake level decrease, or explains 83.5% of the post-TGD annual lake level drop (Auxiliary material, Table S4). These results suggest that extreme climate conditions can dominate the influence of the TGD on Poyang Lake recession, which is similar to the effects of climate on the Changjiang River in 2006^25^.

Meanwhile, variation of evapotranspiration, the sum of evaporation and plant transpiration, may also influence the water level of the Poyang Lake. Compared with the pre-TGD period, monthly evaporation over the Poyang Lake region indicates slight increase in March, April and May, and denotes decrease in the rest 9 months (Auxiliary material, Fig. S7). In October, evaporation over the lake region decreased from 0.12 m during 1970–2002 to 0.11 m during 2003–2012, due to rising air temperature and increasing humidity^26^. Accordingly, Poyang Lake keeps around 0.01 m more water in October in terms of less evaporation with respect to pre-TGD period. Meanwhile, according to Ye *et al.*^27^, the potential evapotranspiration over the Poyang Lake showed long term decreasing trends in summer, autumn and winter during 1960–2008 along with decreasing trends in sunshine duration, wind speed and relative humidity, and increasing trend in air temperature, which coincides with the trend of evaporation. Since vegetation transpiration has no significant contribution to evapotranspiration and water level variation of the Poyang Lake, its influence is ignored in this study.

It is also worth mentioning that the decreased tributary inflow can be caused by decreased basin precipitation as well as increased water utilization. Over the Poyang Lake catchment, yearly water use increases by 15.8 × 10^8 ^m^3^ from 211.1 × 10^8^ m^3^ in pre-TGD stage to 226.9 × 10^8^ m^3^ in post-TGD stage, along with the population growth and economic development (Auxiliary material, Fig. S8). Remove the return part from the water usage cycle, the unreturned water consumption over Poyang Lake basin can result in 0.025 m water level reduction in October. Therefore, the contribution of tributary precipitation reduction to Poyang Lake recession decreased from 0.86 m to 0.835 m. It means that the impact of regional water use variation on lake level reduction is limited.

### Anthropogenic activities in the Poyang Lake basin

The intensive anthropogenic forcing over the Poyang Lake watershed mainly includes sand extraction and dam regulations along the tributaries. By extracting sand directly from the channel bed, sand mining may change the lake channel geometry and induce channel incision^28^,^29^. It is revealed that sand dredging from the Poyang Lake occurred during 2001–2008 and sand was exported at a rate of 236 million m^3^ per year^30^. Here, extrapolating this volume of sand over the 3000 km^2^ surface of the Poyang Lake can result in 0.1 m lowering of the entire lakebed in average, which accounts for 4.4% of the lake level decline. This study use average rather than accumulative variable to represent the effect of sand mining on lake level variation because sand mining over the Poyang Lake primarily created local deep pools and pits, which usually are lower than the lake bed datum (Auxiliary material, Fig. S9). The stored water in these scars thereof are highly likely been recharged by groundwater, even though partly are filled by lake surface water. Moreover, the trapped water in the previous old sand scars are dead water, which rarely participate in the water cycle of the Poyang Lake (Auxiliary material, Fig. S9). Therefore, the lake surface water only partly been affected by the new added scores that are created within the current year. It’s probably that we overestimate the contribution of sand mining to water level reduction of Poyang Lake in this study. Further investigation related to the number of new scars in each year and the incised volume of each scar is needed to accurately analyze the relationship between stored water and lake level.

Meanwhile, there are 25 large and 211 medium-sized reservoirs with a total storage capacity of 27.89 km^3^ located in the Poyang Lake basin at present^31^ (Auxiliary material, Table S2), which constitute a main factor affecting the sediment load from the five sub-basins to the lake. Relative to the pre-TGD period, the annual SSD input was 74.8% lower (from 1290 × 10^4^ t to 577 × 10^4^ t), while the yearly SSD output from the lake to Changjiang River significantly increased, from 922 × 10^4 ^t to 1252 × 10^4 ^t. By comparing the variation between sediment input and output, we find that the lake changed from a depositional to an erosional pattern in 2003 and lost 1043 × 10^4 ^t of sediment annually. However, this lost SSD only causes a 0.004 m lake table decline, which equals 0.2% of the total lake level decline.

### Three Gorges Dam regulation

**Figure 5:**
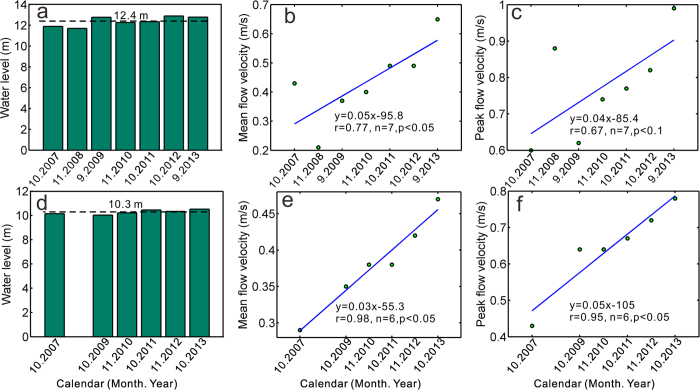
Flow velocity at Hukou station in initial and middle water-falling stages, with (a) water level scenario for initial water-falling stages; (b) mean flow velocity in initial water-falling stages; (c) peak flow velocity in initial water-falling stages; (d) water level scenario for middle water-falling stages; (e) mean flow velocity in middle water-falling stages; and (f) peak flow velocity in middle water-falling stages. The figure was created with Matlab R2009b.

As the largest dam in the world, the TGD caused a 70% reduction of sediment flux into the sea and significant erosion along the middle-lower reach of the Changjiang River^20^,^32^. Along the channel adjacent to Poyang Lake, Jiujiang and Datong stations show an average 1.22 m and 1.04 m of decline in bed level, respectively. On the other hand, Hukou station, in an area that fundamentally determines the lake-river exchange ability, show little morphological change. Compared with the pre-TGD stage, the topographic gradient between Poyang Lake and Changjiang River increased considerably while the water level gradient from upper reaches (Duchang) towards lake mouth (Hukou) has no observable variation in post-TGD period (Auxiliary material, Fig. S10). The significant enlarged water level difference between the Poyang Lake and the Changjiang River, together with the relatively stable water level gradient inside of the Poyang Lake, dramatically increase the lake’s discharge ability and thus lead to lake level decline. This has been clearly reflected by the temporal variation of flow velocity under similar water level scenarios at Hukou station (Fig. 5). Statistical significant upward trends have been detected in both initial and middle stages of lake water-falling during 2007-2013. In initial water-falling stage, when the lake level around 12.4 m (Fig. 5a), the mean and peak flow velocity at Hukou respectively increase from 0.43 m/s in 2007 to 0.65 m/s in 2013 and from 0.6 m/s in 2007 to 0.99 m/s in 2013 (Fig. 5b,c). In middle water-falling stage, when the water level decreases to 10.3 m (Fig. 5d), the mean flow velocity at Hukou increases by 0.18 m/s from 0.29 m/s in 2007 to 0.47 m/s in 2013 while the peak flow velocity increases by 0.35 m/s from 0.43 m/s in 2007 to 0.78 m/s in 2013 (Fig. 5e,f). It is precisely because of the enlarged water level gradient between the Poyang Lake and the Changjiang River, the flow velocity at lake outlet increases. In addition to the stable water level gradient inside of the Poyang Lake, the lake’s outflow increases dramatically and thus accelerates the lake level decline and lake recession.

Meanwhile, the TGD stores water from September to early November, which significantly decreases the downstream discharge. The mean October discharge at Yichang and Datong stations, averaged 18000 m^3^/s and 33100 m^3^/s, respectively, during 1960-2002, which reduced to 12300 m^3^/s and 25600 m^3^/s after 2003. Following dam regulation, the average monthly water level in October dropped by 3.3 m and 1.9 m in Yichang and Datong, respectively, which further increased the water level gradient between the lake and the river as well as the flow velocity, resulting in greater lake outflow (Auxiliary material, Figs S11; S12).

Moreover, we note that the current water velocity at Duchang reaches a maximum when the water level is approximately 12.4 m, which is the demarcation point between a floodplain and a sub-lacustrine channel (Auxiliary material, Fig. S13). Relative to the pre-TGD period, this demarcation water level was reached earlier, in October rather than November, due to TGD regulation. When the overbank flow recedes into the lake channel, the current velocity increases dramatically, which forces a large amount of lake water to enter the river and induces a sharp decrease in lake level.

Besides, when the water level decreases dramatically, groundwater may participate in the water interaction between the Poyang Lake and the Changjiang River to keep the lake water balance^33^. By analyzing the groundwater characteristic at Hukou station, it is found that the contribution of groundwater to total flow in October in pre- and post-TGD periods are respectively 39.1% and 39.6%, indicating 0.5% increase (Auxiliary material, Fig. S14). Such a difference can induce the lake level decrease by 0.03 m, which is relatively small against the 2.24 m lake level reduction.

Hereby we recognize precipitation variation, anthropogenic activities over the Poyang Lake basin and TGD regulation as the main causes for the Poyang Lake shrinkage. Because precipitation variation and anthropogenic activities over the Poyang Lake respectively account for 39.1% and 4.6% of lake decline, the overall impact of the TGD on the Poyang Lake recession can be quantified as 56.3% (Auxiliary material, Table S3). During extreme drought conditions, though, the influence of precipitation variation on lake recession exceeds that of TGD regulation (Auxiliary material, Table S4). When considering the influence of the rest possible impact factors, including evapotranspiration decrease, water consumption increase and groundwater contribution variation, the contribution of precipitation variation, and TGD regulation to the Poyang Lake recession reduce from 39.1% to 37.8% and from 56.3% to 54.9%, respectively while that of human activities over the Poyang Lake catchment goes up from 4.6% to 6%, indicating around 1% fluctuation as a whole (Auxiliary material, Table S5).

## Conclusions

Given its cultural, ecological, and economic significance, the recession of Poyang Lake has received international attention in the context of global lake shrinking, notably because of the exposure of the Ming Dynasty “thousand-eye bridge”. Many complex effects may have contributed to the lake’s recession since the beginning of the 21st century, including TGD regulation, sand mining and climate variation. Our results indicate, firstly, that Poyang Lake has experienced prolonged extreme low water level since 2003 and has been entering its dry season 28 days early, on 16^th^ October instead of 12^th^ November. Channel bed erosion along the middle-lower Changjiang River and extensive impoundment during September and October because of TGD regulation have increased the lake-river water level gradient and thus the lake’s outflow to the Changjiang River. Hence, the distant TGD, approximately 1000 km upstream of Poyang Lake, explains 56.3% of lake level decrease over the past decade, constituting the fundamental forcing for lake recession (Auxiliary material, Table S3). Variations in precipitation and anthropogenic activities over the Poyang Lake catchment are responsible for 39.1% and 4.6% of the lake level decline, respectively, which has exacerbated the problem of lake recession. Besides, consideration of evapotranspiration decrease, water use increase and groundwater contribution variation induces around 1% fluctuation in the quantification estimation. We also note that, in extreme drought conditions, the impact of TGD regulation on Poyang Lake recession can be overruled by climate forcing (Auxiliary material, Table S4).

The recession of Poyang Lake is due to complex and inter-coupled hydrological processes. Without an appropriate policy response, it can be expected that the recession of China’s largest freshwater lake will only worsen in view of the dramatic influence of the TGD on Poyang Lake levels. We argue that the influence of the TGD on Poyang Lake recession should be re-evaluated.

## Methods

### Data sources

Hydrology and morphology data provide direct information on lake evolution over time. Four groups of gauged hydrological and meteorological measurements were used in this study.

The first group includes daily water level, discharge and SSD at Jiujiang, Hukou, Datong, Duchang, Xingzi, Wanjiabu, Waizhou, Lijiadu, Meigang, Hushan and Dufengkeng stations from 1960–2012. Water level is automatically recorded with level-sensing technologies. The monitoring of discharge is mainly carried out using velocity meters while float gauging and stage rating curves are used for some sites. SSD measurement involves sampling, suspended sediment concentration (SSC) estimation and SSC flux generation. Daily hydrologic records for the Changjiang River and the Poyang Lake catchment were available from the Changjiang Water Resources Commission.

The second group of measurements addresses the geomorphic features of lake mouth and the Changjiang River, which were measured at Jiujiang, Hukou and Datong stations from 1965–2012 using ultra-sonic depth finder and theodolite. The number and location of sampling points depends on the section width. These data were obtained from the Changjiang Water Resources Commission.

The third group of measurements is daily precipitation and daily evaporation data from the Poyang Lake catchment. The daily precipitation were collected by 20 cm caliber JDZ rain gauges while the daily evaporation were measured by 20 cm caliber pan-evaporator. The precipitation data, covering a time period from 1960 to 2012, were downloaded from the National Climatic Centre of the Chinese Meteorological Administration. The evaporation date, covering a time period from 1970 to 2012, were obtained from the Changjiang Water Resources Commission.

Additionally, lake current information at Duchang station and flow velocity information at Hukou station, been measured with current meters and ADCP (Acoustic Doppler Current Profiler) technique, were acquired from the Changjiang Water Resources Commission.

The measurement frequency for each hydrological parameter depends on the weather. Normally, more observations are obtained during the wet season. All of the measurements strictly follow the relevant national standards.

### Methods

Statistical methods provide an effective way of identifying physical characteristics from the observations. In this study, methods of moments are used to assess the population parameters. Linear and quadratic polynomials are developed to detect the relationship between water level and discharge at Hukou, and relate the water level of Xingzi with that of Dudang. Goodness of fit is tested by correlation coefficients. Sen’s slope test is applied to detect whether the differences in water level between the pre- and post-TGD periods at Duchang from September 1^st^ to November 31^st^ has a significant tendency and to determine the true slope of the trend^34^,^35^ (Auxiliary material). The standard normal homogeneity test is applied to the water level time series for the same day of every year at Duchang from 1960–2012 to identify the occurrence of abrupt changes^36^ (Auxiliary material). Grouped frequency distribution is used to generate the relative frequencies and relative cumulative frequencies of daily water level at Duchang and Hukou. Water level at Duchang is categorized into 8 groups with an interval of 2 m while water level at Hukou is classified into 9 groups with the same interval. The topographic gradient between the Changjiang River and the Poyang Lake is calculated between Jiujiang and Hukou and between Hukou and Datong. For each station, the mean elevation of the channel bed is calculated to infer channel depth. The topographic gradient between two stations is calculated as the ratio between their channel depth difference and distance. The measured daily evaporation is transferred into lake surface evaporation through instrument-conversion method^26^. Flow velocity under similar lake level scenarios during 2007–2013 are analyzed with linear regression method. For the sake of comparison, we focus on both initial and middle water-falling stages during September-November in this study (Auxiliary material, Table S6).

The effect of inflow, anthropogenic activities and groundwater on lake shrinkage is quantified in terms of water level. For sand mining, we convert the sediment volume into water volume, and then spread the water volume on top of the 3000 km^2^ surface area to obtain a corresponding water level. For sediment variation, we calculate the lake sediment budget between input and output to estimate whether the lake falls into an erosional or a depositional pattern and then transform this budget to water level. For tributary inflow, water use and groundwater, we set the variable of discharge as a frustum (Auxiliary material, Fig. S15). For a given surface area, expressing the bottom area as a function of water level, the relevant water level can be determined by solving the frustum volume formula (Auxiliary material).

## Additional Information

**How to cite this article**: Mei, X. *et al.* Linkage between Three Gorges Dam impacts and the dramatic recessions in China,s largest freshwater lake, Poyang Lake. *Sci. Rep.* **5**, 18197; doi: 10.1038/srep18197 (2015).

## Supplementary Material

Supplementary Information

## Figures and Tables

**Figure 1:**
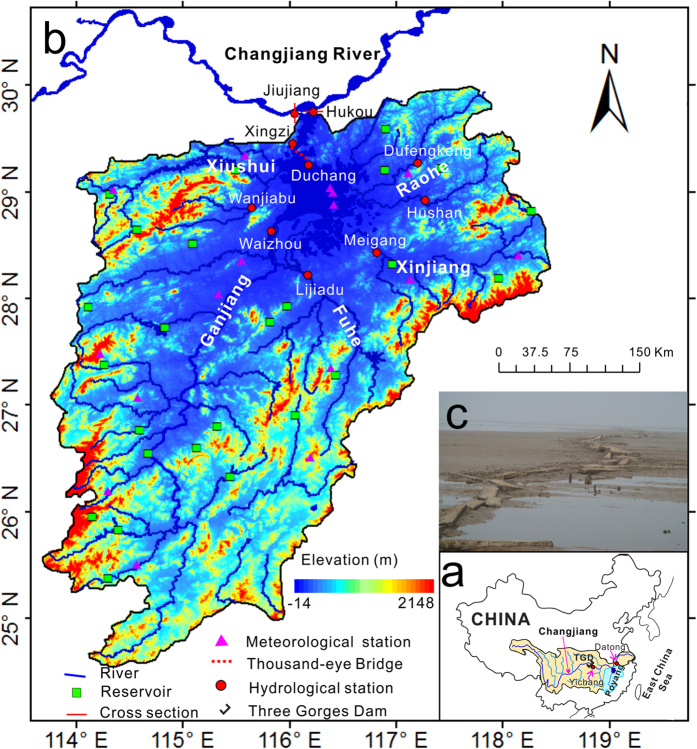
Map showing the Poyang Lake catchment, with (a) Poyang Lake’s location in relation to the Changjiang River, the Three Gorges Dam and the East China Sea; (b) the Poyang Lake catchment; and (c) the “thousand-eye bridge” built during the Ming dynasty (photo taken at Duchang on 3 January, 2015). The map was created with ArcGIS10.1.
