# Supplementary material for: Linkage between Three Gorges Dam impacts and the dramatic recessions in China’s largest freshwater lake, Poyang Lake
Source: Sci Rep. 2015 Dec 11;5:18197. doi: 10.1038/srep18197 (PMC4675997; doi:10.1038/srep18197)
Supplement: Supplementary Information [file srep18197-s1.pdf]

Supplementary Information for:

Linkage between Three Gorges Dam impacts and the dramatic recessions in China's largest freshwater lake, Poyang Lake

Supplementary data

Poyang Lake Catchment

As a typical throughput lake, Poyang Lake (28.37° -29.75° N and 115.78° -116.75° E) mainly receives water from the Ganjiang, Fuhe, Xinjiang, Xiushui and Raohe Rivers, and discharges water to the Changjiang (Yangtze) River at Hukou. The 766-km long Ganjiang River is the largest tributary to the Poyang Lake in terms of sediment load and discharge, followed by the Xinjiang and Fuhe Rivers. In this study, the discharge and sediment load from the Ganjiang, Fuhe and Xinjiang Rivers are measured at the main stream, while measurements for the Xiushui and Raohe Rivers are recorded at upstream tributaries (Table S1).

Supplementary methods

Grouped frequency distribution

Grouped frequency distribution is used to detect the statistical characteristics of a large number of continuous variables. The technique, groups values into intervals according to their amplitude and assigns each interval to a frequency. The grouped frequency can be expressed as either relative frequency or relative cumulative frequency.

The relative frequency is defined as:

$$PF = \frac{n_i}{n} \qquad \text{(S1)}$$

where n_i is the number of observations that occur in a certain class, and n is the total number of observations.

The relative cumulative frequency is set as the quotient between the sum of all of the classes that are less than or equal to the one under consideration and the total number of observations. In this study, grouped frequency distribution is used to generate the relative frequencies and relative cumulative frequencies of daily water levels at Duchang and Hukou (Fig. 2; Fig. 3; Fig. S3). Water level at Duchang is categorized into 8 groups with an interval of 2 m while water levels at Hukou are classified into 9

groups with the same interval.

Sen's slope estimator

Sen's slope estimator accounts for the magnitude of trend in a sample of N pairs of data through a non-parametric procedure[1]:

$$Q_i = \frac{x_j - x_k}{j - k} \qquad i = 1, \cdots, N \tag{S2}$$

Where x_j and x_k are measurements at times j and k (j>k), respectively. The median of Sen's slope estimator is computed by:

$$Q_{med} = \begin{cases} Q_{[(N+1)/2]} & N \text{ is odd} \\ \dfrac{Q_{[N/2]} + Q_{[(N+2)/2]}}{2} & N \text{ is even} \end{cases} \tag{S3}$$

The sign of Q_{med} reflects the direction of the trend while the value of Q_{med} shows the steepness of the trend. To test whether the median slope is significantly different than 0, Q_{med} is tested with a two-sided test at the 100(1-α) % confidence interval[2]. In this paper, Sen's slope test is adopted to detect whether the time series of water level differences between pre- and post-TGD periods at Duchang from 1st September to 31st November has a significant tendency, as well as the true slope of the trend (Fig.S2 a).

Standard normal homogeneity test

The standard normal homogeneity test (SNHT) identifies the occurrence of abrupt change by comparing the mean of the first *a* years of the record with that of the last *n*-*a* years based on the statistic T_0[3]:

$$T_0 = \max_{1 \le a < n} T_{(a)} = \max_{1 \le a < n} (a\bar{z}_1^2 + (n-a)\bar{z}_2^2), a = 1, 2, \ldots, n \tag{S4}$$

where

$$\bar{z}_1 = \frac{1}{a} \sum_{i=1}^{a} \frac{(Y_i - \bar{Y})}{s}; \quad \bar{z}_2 = \frac{1}{n-a} \sum_{i=a+1}^{n} \frac{(Y_i - \bar{Y})}{s} \tag{S5}$$

$\bar{Y}$ is the mean value of the data set, *s* is the standard deviation of the data set, and *n* is the length of the data set.

When T_0 approaches the maximum value at year $a=A$, a significant shift occurs if T_0 is larger than the critical value at a given significance level. Here, SNHT is applied to the time series of water level for the same day of every year at Duchang from 1960-2012 to identify the occurrence of abrupt changes (Fig.S2b).

Water level estimation

When calculating the variable of inflow-induced water level variation, we assumed the Poyang Lake as a frustum (Fig. S15). The formula for a frustum volume is:

$$V = \frac{1}{3}H(S' + S + \sqrt{S'S}) \qquad (S6)$$

where S' is the area of the upper base, S is the area of the lower base, and H is the height. Because the volume and the upper base area of the frustum are known, the area of the bottom base can be estimated as a function of H, and H can then be determined by solving Equation S6.

Definition of extreme drought events

The low-frequency of rare events is often defined by a particular high or low quantile[4]. In this study, monthly October precipitation over the Poyang Lake during 1960-2012 is categorized on the basis of the 5% quantile. Extreme drought conditions correspond to the precipitation below the 5% quantile. Accordingly, 2004 is determined as an extreme drought year over the Poyang Lake basin.

Quantitative analysis of precipitation variation, anthropogenic activities in the Poyang Lake basin and TGD regulation on Poyang Lake recession

The variation of Poyang Lake's water level between pre- and post-TGD stages can be expressed via the linear equation:

$$\Delta h = h_p + h_e + h_a + h_T + h_g \qquad (S7)$$

where h_p, h_e, h_a, h_T, and h_g represent the variation of lake water level in response to precipitation variation, evapotranspiration variation, anthropogenic activities in the Poyang Lake basin ,TGD regulation and groundwater variation, respectively. The effect of evapotranspiration variation, groundwater variation and regional water consumption

variation on lake level decline are ignored firstly due to their limited effect, and then are further discussed as follows to avoid possible uncertainties.

Anthropogenic activities. Human activities in the Poyang Lake basin are mainly sand mining and dam operation.

Sand from Poyang Lake was exported at a rate of 236 million m^3 pear year from 2001-2008[5]. Averaging this amount of sand over the total lake area of 3000 km^2, the entire lake level will be almost 0.1 m lower in average with respect to pre-TGD period, which explains 4.4% of lake level decline. We use average rather than accumulative variable to represent the effect of sand mining on lake level variation in this study for the following reasons: 1) Sand mining over the Poyang Lake primarily created local deep pools and pits, which usually are lower than the lake bed datum (Fig. S9). The stored water in these scars thereof are likely mainly been recharged by groundwater and partly been filled by lake surface water, which would not generate entire lake decline; 2) The trapped water in the previous old sand scars are dead water, which rarely been measured or participate in the water cycle of the Poyang Lake even in low lake level scenario (Fig. S9). Therefore, the lake surface water only partly been affected by the new added scores that are created within the current year. It's probably that we overestimate the contribution of sand mining to water level reduction of Poyang Lake in this study. Further investigation related to the number of new scars in each year and the incised volume of each scar is needed to accurately analyze the relationship between stored water and lake level.

The Poyang lake catchment is controlled by 25 large and 211 medium sized reservoirs (Table S2), which significantly affect sediment input to the lake region. Annual gross sediment loss due to upstream reservoir operation is 1043×10^4 t during 2003-2012, which corresponds to a 0.004 m lowering every year and accounts for 0.2% of lake level decline.

Regional water consumption is another possible factor affecting the lake level variation. Poyang Lake, covering 97% of the area of Jiangxi Province, is the main water resource supplier of Jiangxi Province[6]. Over 90% of water resources of Jiangxi Province comes from the Poyang Lake (Poyang Lake region, 20.4%; Poyang Lake

basin, 70.6%)[7]. Therefore, the water consumption of Jiangxi Province can represent the situation of water use over the Poyang Lake catchment. The yearly water consumption, including industry, agriculture and urban use of Jiangxi Province during 2000-2012, is shown in Fig. S8. It is found that water use of Jiangxi Province increases by 15.8×10^8 m^3 from 211.1×10^8 m^3 in pre-TGD period to 226.9×10^8 m^3 in post-TGD period along with the population growth and economic development. Among them, 45% are unreturned water[8-9]. Since the highest water use occurs during July to September in Jiangxi Province, this study estimates the water use variation in October by the average variable: divide the yearly increased unreturned water by 12. Accordingly, increased regional water consumption leads to a 0.032 m water level reduction in October, among which 0.007 m is caused by lake region water use, 0.025 m is caused by lake basin water use.

In short, the contribution of human activities to the water level reduction of the Poyang Lake is 4.6%, which increases to 6% when regional water consumption variation is considered.

Climate change. Changes in precipitation is the most critical factor determining the impact of climate change. Precipitation affects the Poyang Lake catchment through two ways. Precipitation over the lake region affects lake level directly. Precipitation over the lake basin affects the lake via received inflow. Relative to the pre-TGD stage, precipitation over the lake region decreased by 0.015 m, which equals 0.7% of total lake level decrease. The contribution of lake catchment inflow decreased by 14.2×10^8 m^3 (from 45.27×10^8 m^3 to 31.07×10^8 m^3) in October, which results in a water level drop of 0.86 m over the entire lake and accounts for 38.4% of lake level decline. The upstream tributary inflow variation can be explained by two factors: precipitation and water use. Since the effect of water use has been quantified as 0.025 m, the rest 0.835 m is resulted from precipitation variation, which accounts for 37.1% of lake level decline. In the case of the extreme drought in 2004, the lake region received 0.06 m less precipitation and 23.9×10^8 m^3 less inflow, which contributed 2.7% and 83.5% of the post-dam annual lake decline, respectively. Since the water use in 2004 is even lower than pre-TGD period (Fig. S8), the decreased tributary inflow is totally explained by

precipitation variation. This results implies that the impacts of TGD regulation on lake shrinkage can be overshadowed by extreme drought.

Evapotranspiration is another important factor reflecting the impact of climate change. The average monthly evaporation is shown in Fig. S7. Evaporation over the lake region decreased from 0.12 m during 1970-2002 to 0.11 m during 2003-2012 in October, due to rising air temperature and increasing humidity[10]. Accordingly, Poyang Lake keeps around 0.01 m more water in October in terms of less evaporation. Due to lack of long-term of observations, this paper evaluates the influence of evapotranspiration on the Poyang Lake water level by quoting the results of other researches. According to Ye et al.[11], the potential evapotranspiration over the Poyang Lake showed long term decreasing trends in summer, autumn and winter during 1960-2008 along with decreasing trends in sunshine duration, wind speed and relative humidity, and increasing trend in air temperature. Since vegetation transpiration has no significant contribution on evapotranspiration and water level variation over the Poyang Lake, its influence is ignored in this study.

Therefore, the contribution of precipitation on Poyang Lake shrinkage is 39.1%, which decreased to 37.8% when regional water use and evaporation are taken into account.

Three Gorges Dam regulation. TGD-induced downstream riverbed erosion increases the topographic gradient between the Poyang Lake and the Changjiang River, which increases the lake's outflow ability, discharging a huge amount of water into the river. Moreover, the filling of reservoirs from September to early November further heightens the lake-river gradient and lake outflow. Because climate change, human activities in the lake basin and TGD operation are the three major effects on the water level variation of the Poyang Lake, the influence of the TGD on lake recession can be quantified as the difference between Δh and the sum of h_p and h_a. Therefore, the TGD is responsible for 1.26 m and 56.3% of lake recession in October. We note, though, that during extreme drought conditions, the impact of TGD regulation on Poyang Lake recession can be smaller than the effects of climate forcing.

Moreover, when the water level decreases dramatically, groundwater may participate

in the water interaction between the Poyang Lake and the Changjiang River to keep the lake water balance[12].Baseflow, as the net flow from groundwater storage to a stream, is a common way to estimate the groundwater characteristic[13]. The digital filtering method is applied to separate baseflow (groundwater) from total flow at Hukou station, and thus to identifies the groundwater infiltration from the Poyang Lake to the Changjiang River[14]. The monthly runoff and groundwater in October are shown in Fig. S14. Compared with pre-TGD stage, the monthly runoff in October decreased by 12% from 107.4×10^8 m^3 to 94.3×10^8 m^3 while the groundwater decreased by 11% from 41.9×10^8 m^3 to 37.3×10^8 m^3. It is shown that the contribution of groundwater to discharge from Poyang Lake to the Yangtze River increased by 0.5% from 39.1% during pre-dam period to 39.6% during post-dam period. Such a difference indicates that groundwater contribute 0.03 m water level reduction in the Poyang Lake recession.

All in all, TGD induced downstream riverbed erosion and water storage explains 56.3% of the Poyang Lake recession. However, this contribution decreases to 54.9% in view of the effect of groundwater, water use and evapotranspiration.

Supplementary tables

Table S1 Flux characteristics of Poyang Lake inflow and outflow

River	Gauging station	Drainage area (km^2)	Mean annual discharge(10^8 m^3)	Mean sediment load (10^4 t)
Ganjiang R.	Waizhou	80948	683	861
Fuhe R.	Lijiadu	15811	127.6	139
Xinjiang R.	Meigang	15535	179.7	206
Raohe R.	Dufengken			
	Hushan	6474	71.19	56.4
Xiushui R.	Wanjiabu	3548	34.9	35.7
Poyang Lake	Hukou	162200	1500	1030

Note: Values given are mean annual hydrological parameters for the period 1950-2010.

Table S2 Description of the major reservoirs in the Poyang Lake catchment

No.	Reservoir	River	Lat.	Long.	Purpose	Finish Year	Capacity (10^8 m^3)
1	Feijiantan	Ganjiang	114.12	27.92	FC,I,H	1960	1.01
2	Daduan	Xiushui	114.57	28.65	FC,I,N	1990	1.15
3	Shangyoujiang	Ganjiang	115.10	28.52	FC,I,H	1960	1.35
4	Ziyunshan	Lakeside	115.82	27.78	FC,I,H	1960	1.2
5	Panqiao	Lakeside	115.98	27.93	FC,I,H	1960	0.74
6	Jiangkou	Ganjiang	114.83	27.73	FC,I,WS	1964	3.46
7	Communism	Raohe	117.43	29.22	FC,I,H	1960	0.83
8	Dongjin	Xiushui	114.32	28.98	FC,I,H	1995	5.61
9	Zhelin	Xiushui	115.50	29.21	FC,I,H,N	1975	50.17
10	Jiepai	Xinjiang	116.97	28.32	N,FC,H	1998	0.51
11	Da'ao	Xinjiang	117.96	28.19	FC,H,I	2000	2.76
12	Qiyi	Xinjiang	118.27	28.82	I,FC,H	1960	2.49
13	Junmin	Lakeside	116.91	29.59	I,FC,H	1972	1.89
14	Bintian	Raohe	116.90	29.21	I,FC,WS	1960	1.15
15	Hongmen	Fuhe	116.43	27.28	H,FC,I	1969	5.42
16	Shangyoujiang	Ganjiang	114.40	25.83	H,FC,N	1957	7.21
17	Youluomen	Ganjiang	114.30	25.38	H,FC,WS	1981	0.86
18	Longtan	Ganjiang	114.15	25.95	H,FC	1996	1.06
19	Tuanjie	Ganjiang	116.06	26.91	H,FC,I	1971	1.02
20	Changgang	Ganjiang	115.45	26.33	H,FC,I	1970	2.51
21	Wan'an	Ganjiang	114.68	26.55	FC,I,H	1990	11.16
22	Laoyingpan	Ganjiang	115.13	26.60	FC,I,H	1983	0.77
23	Sheshang	Ganjiang	114.27	27.38	FC,I,H	1973	1.43
24	Baiyunshan	Ganjiang	115.32	26.80	FC,I,H	1969	0.9
25	Nanche	Ganjiang	114.60	26.77	FC,I,H	1999	1.23

Note: FC is flood control structure; WS is water supply; H is hydroelectric; I is irrigation; and N is navigation.

Table S3 Quantification of various forcings on the Poyang Lake recession

Parameter		Pre-TGD	Post-TGD	Variation	Δh (m)	Contribution
Lake water level		14.67	12.43	-2.24	-2.24	100%
1	Lake region	0.063 m	0.048 m	-0.015 m	-0.015	0.7%
	Lake basin	45.3×10^{8} m^3	31.1×10^{8} m^3	-14.2×10^{8} m^3	-0.86	38.4%
2	Sand mining		2.36×10^{8} m^3/yr	-2.36×10^{8} m^3	0.098	4.4%
	Sediment	368×10^{4} t	-674×10^{4} t	-1043×10^{4} t	-0.004	0.2%
3					-1.26	56.3%

Note: Lake level is measured at Duchang station. Δh is water level variation; 1 is precipitation variation; 2 is anthropogenic activities; and 3 is TGD regulation.

Table S4 Quantification of extreme drought on the Poyang Lake recession

Parameter	Pre-TGD	Post-TGD	Variation	Δh (m)	Contribution
Lake water level	14.67 m	12.43 m	-2.24 m	-2.24	100%
Lake region	0.063 m	0.001 m	-0.06 m	-0.06	2.7%
Lake basin	45.3×10^{8} m^3	21.4×10^{8} m^3	-23.9×10^{8} m^3	-1.87	83.5%

Table S5 Quantification of all possible forcings on the Poyang Lake recession

Parameter		Pre-TGD	Post-TGD	Variation	Δh (m)	Contribution
Lake water level		14.67	12.43	-2.24	-2.25	100%
Evaporation		0.12	0.11	0.01		
1	Lake region	1.61×10^{8} m^3	1.73×10^{8} m^3	0.12×10^{8} m^3	-0.007	0.3%
	Lake basin	5.59×10^{8} m^3	6.01×10^{8} m^3	0.42×10^{8} m^3	-0.025	1.1%
2	Lake region	0.063 m	0.048 m	-0.015 m	-0.015	0.7%
	Lake basin	45.3×10^{8} m^3	31.1×10^{8} m^3	-14.2×10^{8} m^3	-0.835	37.1%
3	Sand mining		2.36×10^{8} m^3/yr	-2.36×10^{8} m^3	0.098	4.4%
	Sediment	368×10^{4} t	-674×10^{4} t	-1043×10^{4} t	-0.004	0.2%
4	Groundwater	39.1%	39.6%	0.5%	-0.03	1.3%
5					-1.24	54.9%

Note: 1 is water use variation; 2 is precipitation variation; 3 is anthropogenic activities; 4 is groundwater variation and 5 is TGD regulation.

Table S6 Time of flow velocity measurement at Hukou Staion during initial and middle water-falling stages

Stage	Year	Date
Initial water-falling	2007	17th October
	2008	5th November
	2009	27th September
	2010	2nd November
	2011	4th October
	2012	11th October
	2013	5th September
Middle water-falling	2007	26th October
	2009	10th October
	2010	12th November
	2011	13th October
	2012	11th November
	2013	17th October

Supplementary Figures

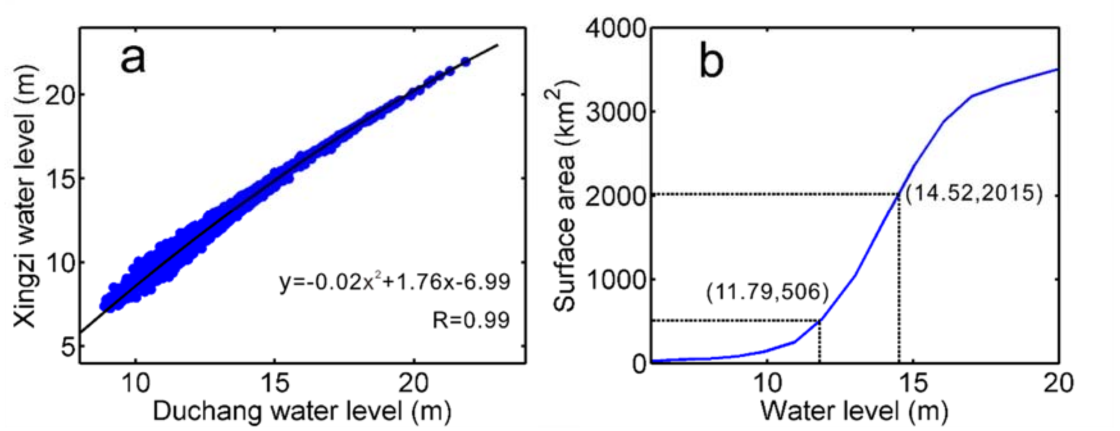

Fig. S1. a) Correlation between water levels at Xingzi and Duchang stations; b) The relationship between lake surface area and water level at Xingzi station. When the water level at Duchang decreases from 16.47 m to 12.43 m, the water level at Xingzi ranges from 14.52 m to 11.79 m, and the lake surface area shrinks from 2015 km^2 to 506 km^2. The figure was created with Matlab R2009b.

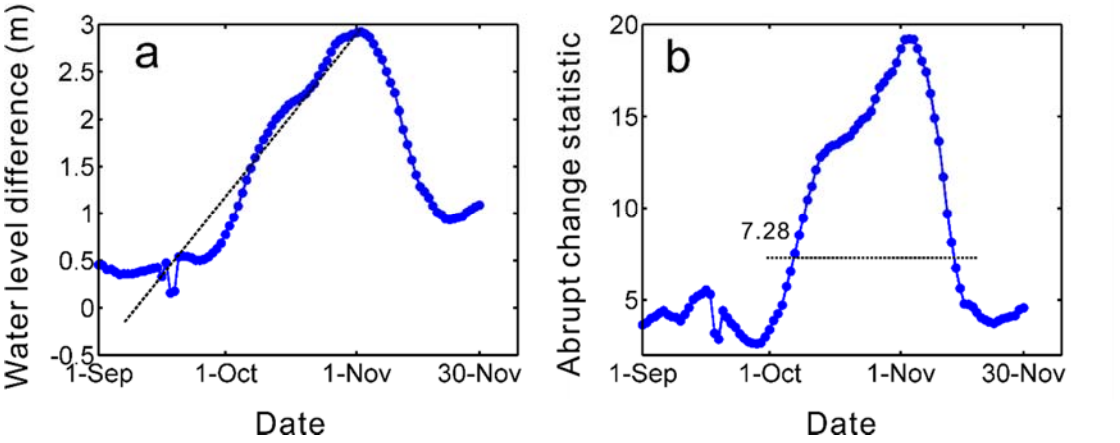

Fig. S2. a) Analysis of trend for the time series of water level differences at Duchang between the pre and post- TGD periods from September 1st to November 31st; b) Abrupt change test for the time series of water level on the same day of every year at Duchang from 1960-2012. The mean values from the pre- and post-TGD periods at each date from September 1th to November 30th are calculated, and then the difference between them is shown in a). Note that the time series of the TGD-induced water level difference has a significant upward trend with a slope of 0.055 from September 7th to November 2nd. The standard normal homogeneity test statistic indicates a sudden change around 2003 when the critical value of 7.28 is passed (5% significant level) on October 7th till November 13th, with a time length of 38 days. Generally, Poyang Lake enters the dry season on November 12th with a water level of 12.6 m at Duchang (12 m at Xingzi); however, the initiation of the dry season moved forward to 16th October in the post-dam period. Also note that the time series of water level on October 16th from 1960-2012 indicates a sudden change in 2003, suggesting that TGD regulation affected the lake's hydrological environment and caused the lake enter the dry season 28 days ahead of normal. The figure was created with Matlab R2009b.

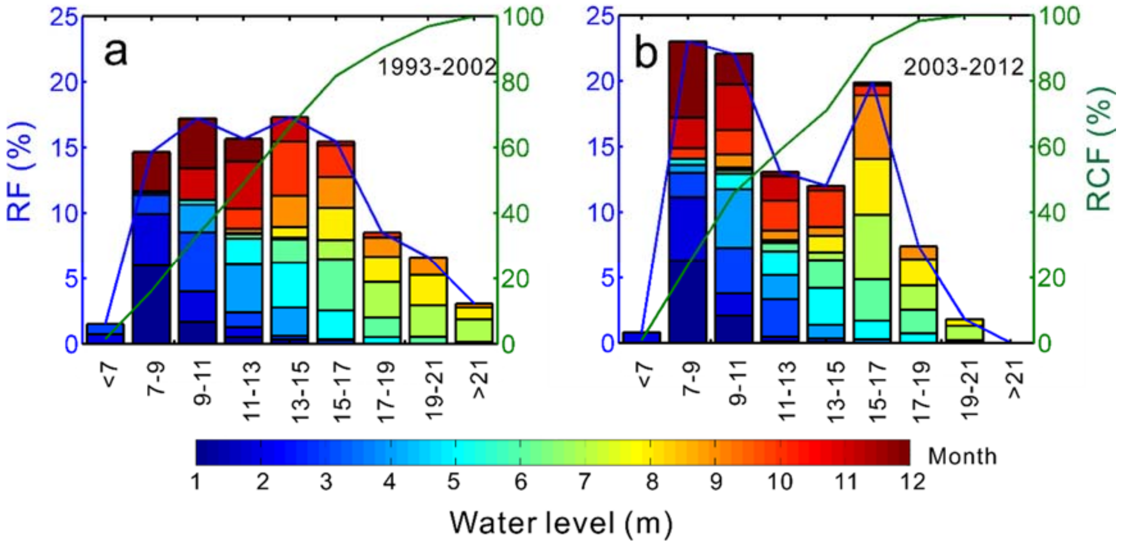

Fig. S3. Occurrence probability of water level at Hukou. The grouped frequency variability of daily water level at Hukou follows that of Duchang, with more water levels below 9 m and fewer water levels above 19 m. The figure was created with Matlab R2009b.

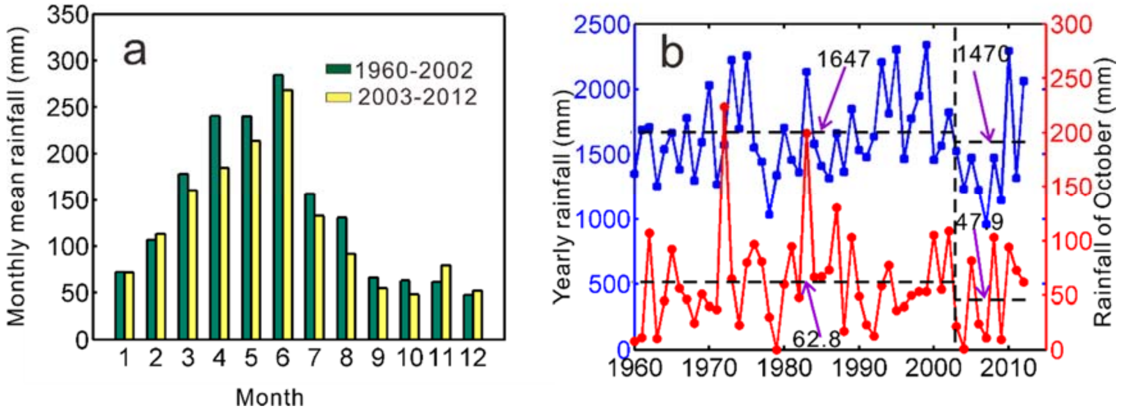

Fig. S4. Precipitation variation from 1960-2012 over Poyang Lake. The monthly precipitation in October of 2004 corresponds to the 3.7% quantile, so it is accordingly defined as an extreme drought event. The figure was created with Matlab R2009b.

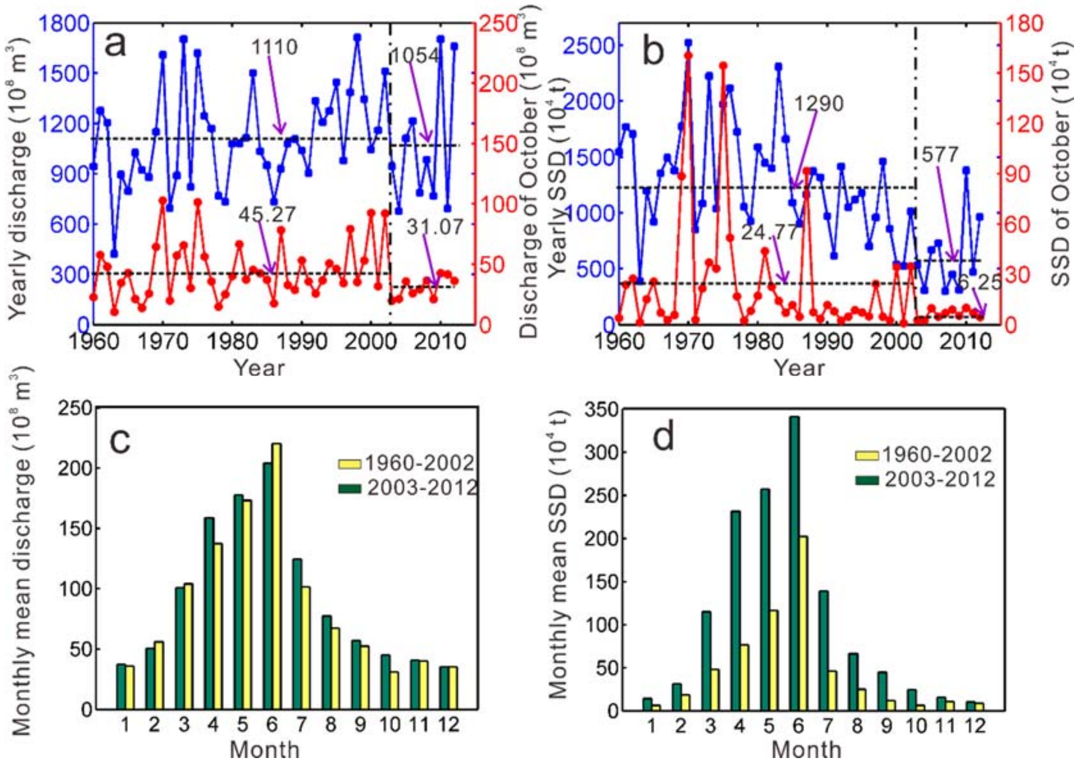

Fig. S5. Flux input of discharge and sediment to Poyang Lake. The figure was created with Matlab R2009b.

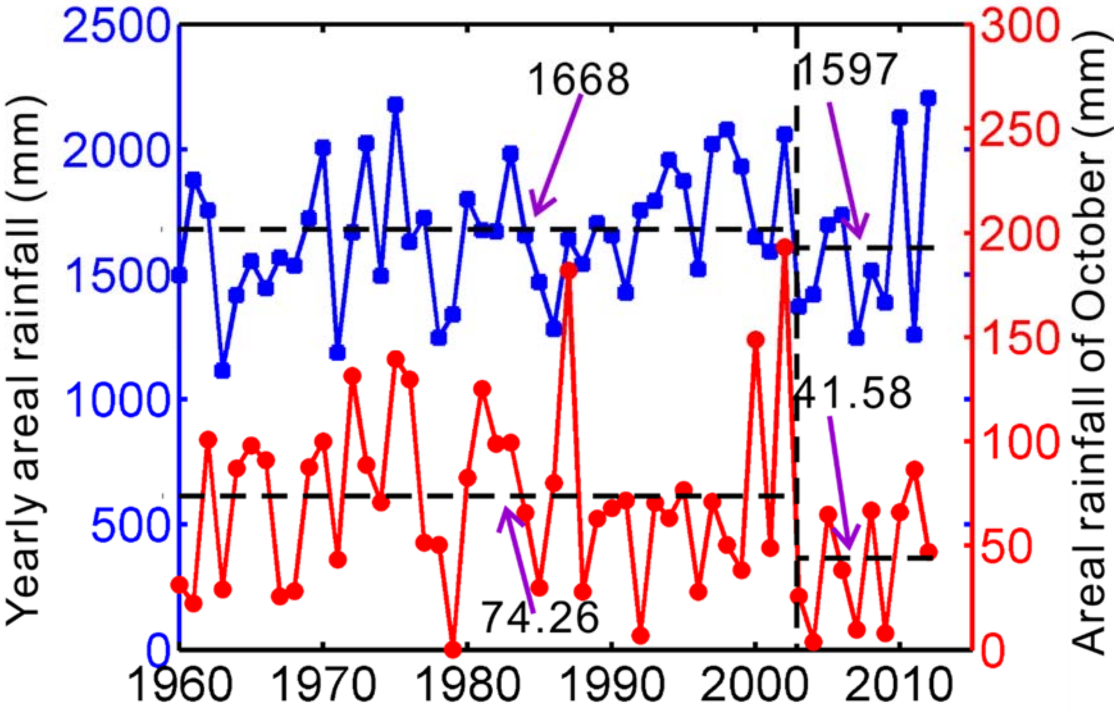

Fig. S6. Precipitation variation from 1960-2012 over the Poyang Lake catchment. Similar to Poyang Lake itself, the lake basin experienced serious drought in October of 2004. Because precipitation in October of 2004 corresponds to the 3.7% quantile, it is recognized as an extreme drought event. The figure was created with Matlab R2009b.

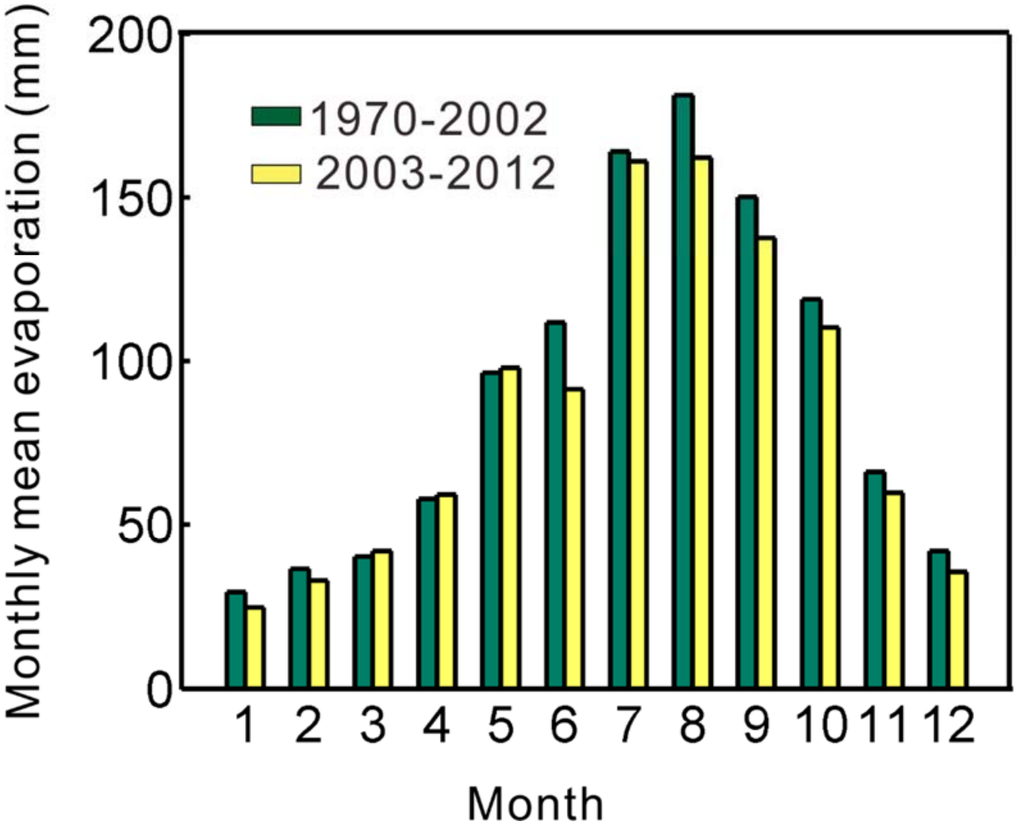

Fig. S7. Evaporation variation during 1970-2012 over the Poyang Lake. The figure was created with Matlab R2009b.

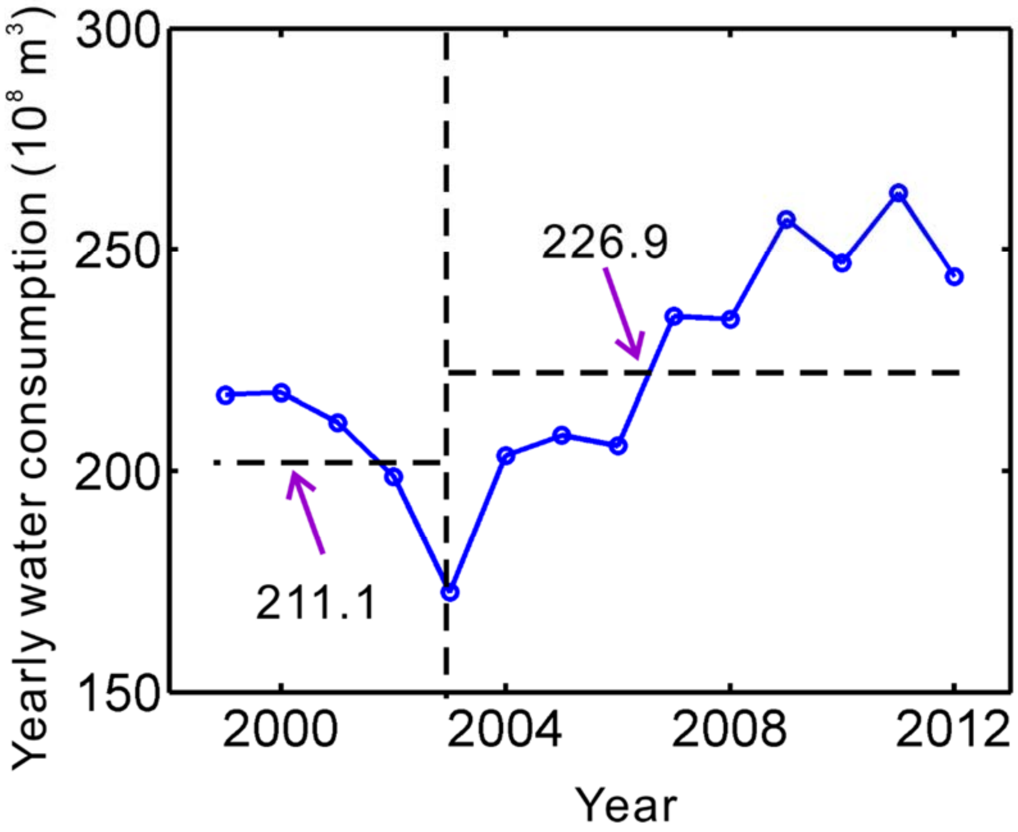

Fig. S8. Yearly water consumption in Jiangxi Province during 1999-2012. The figure was created with Matlab R2009b.

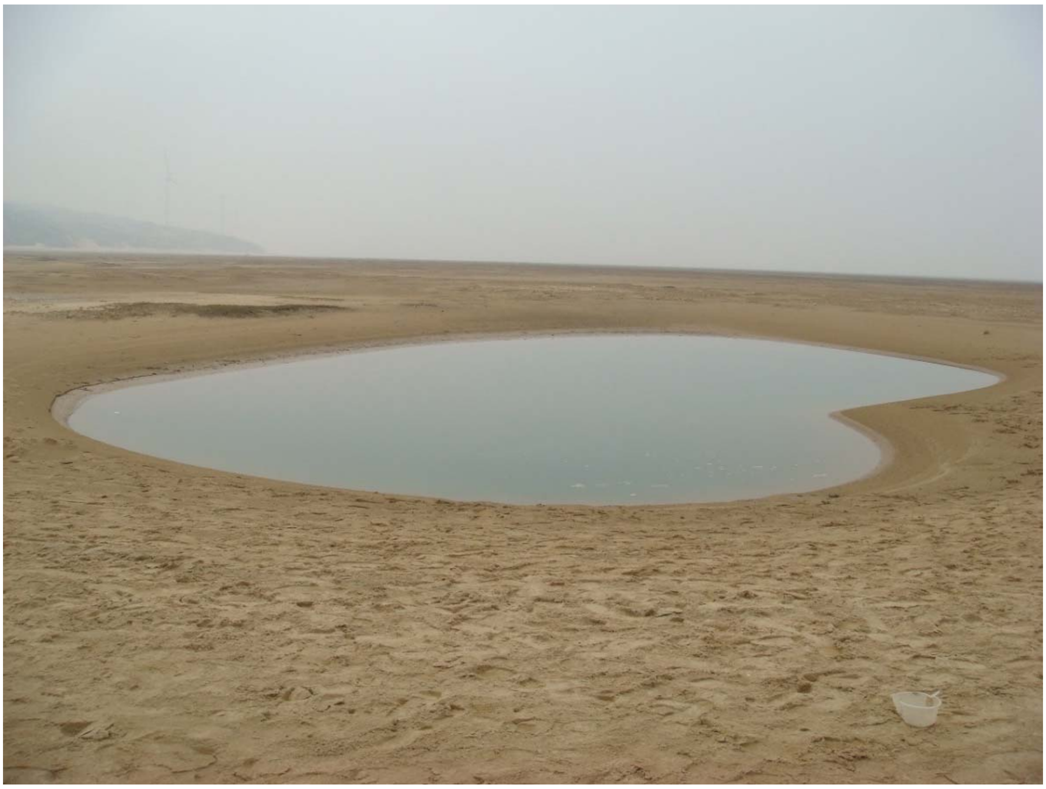

Fig. S9. Sand mining-induced scars in the lake (photo taken at Duchang on 3 January, 2015).

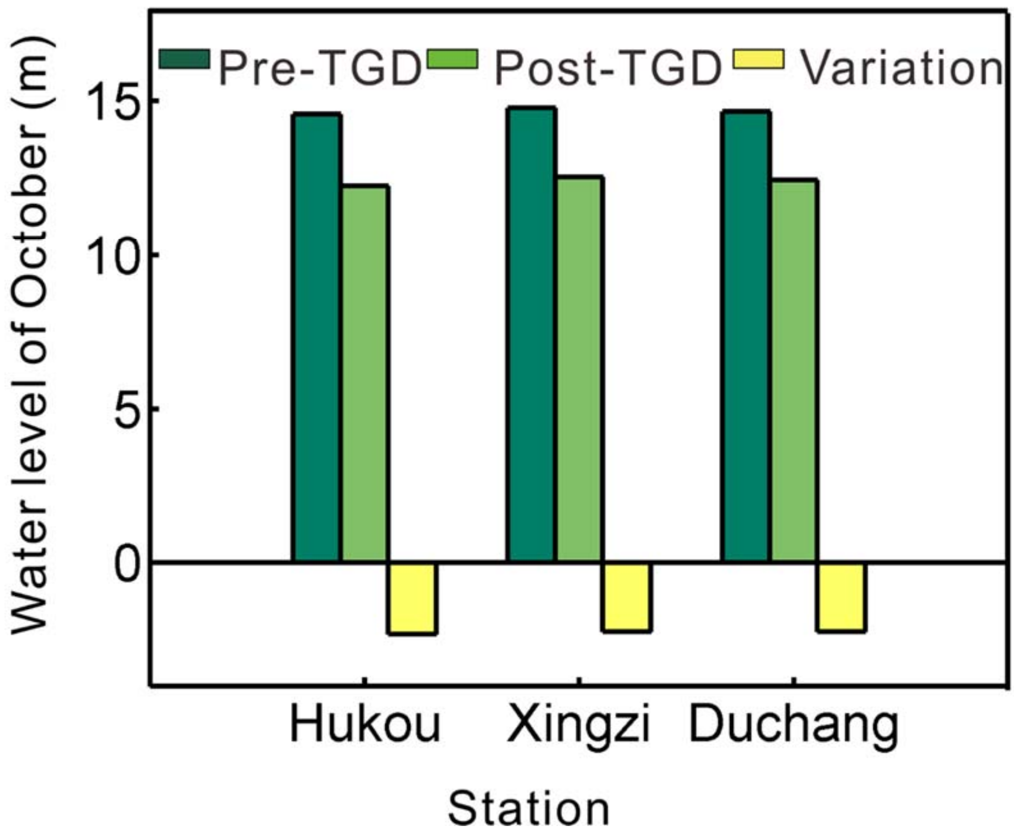

Fig. S10. Water level reduction over the Poyang Lake in Octocer. From lake outlet towards the upper reaches of the lake, the water level reduction in October are 2.32m (Hukou), 2.24 m (Xingzi) and and 2.24 m (Duchang), respectively. Although there is a slight decrease trend in water level reduction from lake outlet to the upper reaches, water level reduction in the three station are still comparable. Moreover, Duchang station is locate in the central area of the Poyang Lake. Therefore, water level reduction of 2.24 m at Duchang station is selected to represent the average water level reduction in the whole lake region. The figure was created with Matlab R2009b.

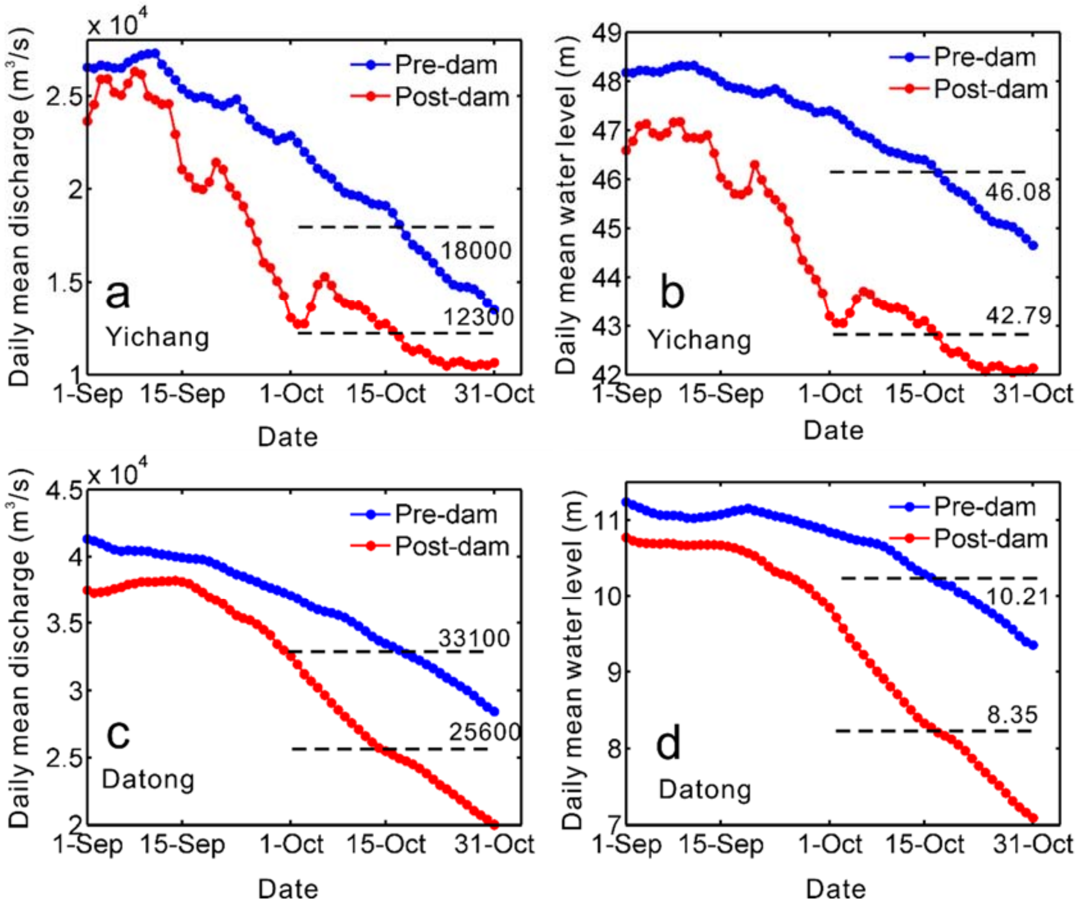

Fig. S11. The mean values of daily discharge and water level in pre- and post-TGD stages from September 1st to October 31st at Yichang (38 km downstream from the TGD) and Datong (1177 km downstream from the TGD). Significant reductions have occurred at both stations since filling of the TGD. The figure was created with Matlab R2009b.

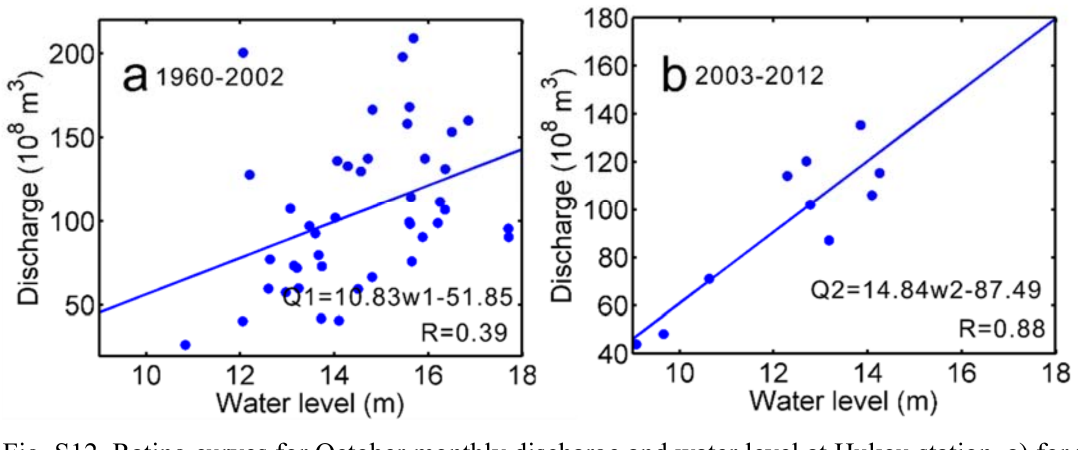

Fig. S12. Rating curves for October monthly discharge and water level at Hukou station, a) for the pre-TGD period; and b) for the post-TGD period. October monthly discharge and water level have a much stronger relationship in the post-TGD period than in the pre-TGD period because TGD operations weakened the blocking effect of the Changjiang River on Poyang Lake. The figure was created with Matlab R2009b.

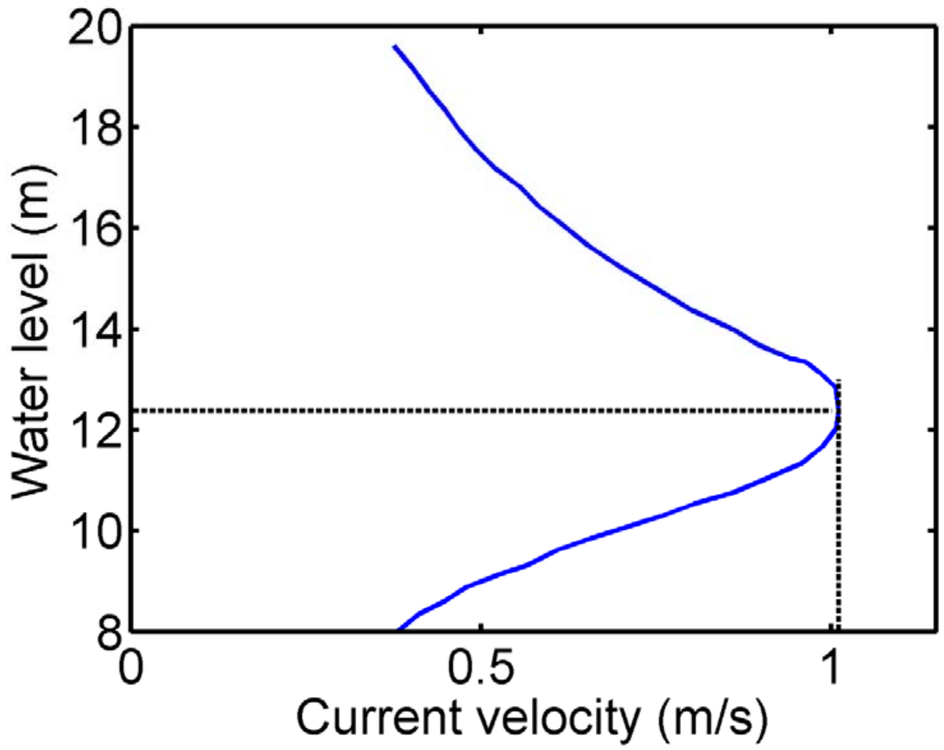

Fig. S13. Relationship between water level and current velocity at Duchang station (Source: Tan et al., 2013)[15]. Current velocity reaches a maximum value when the water level approaches 12.38 m. The figure was created with Matlab R2009b.

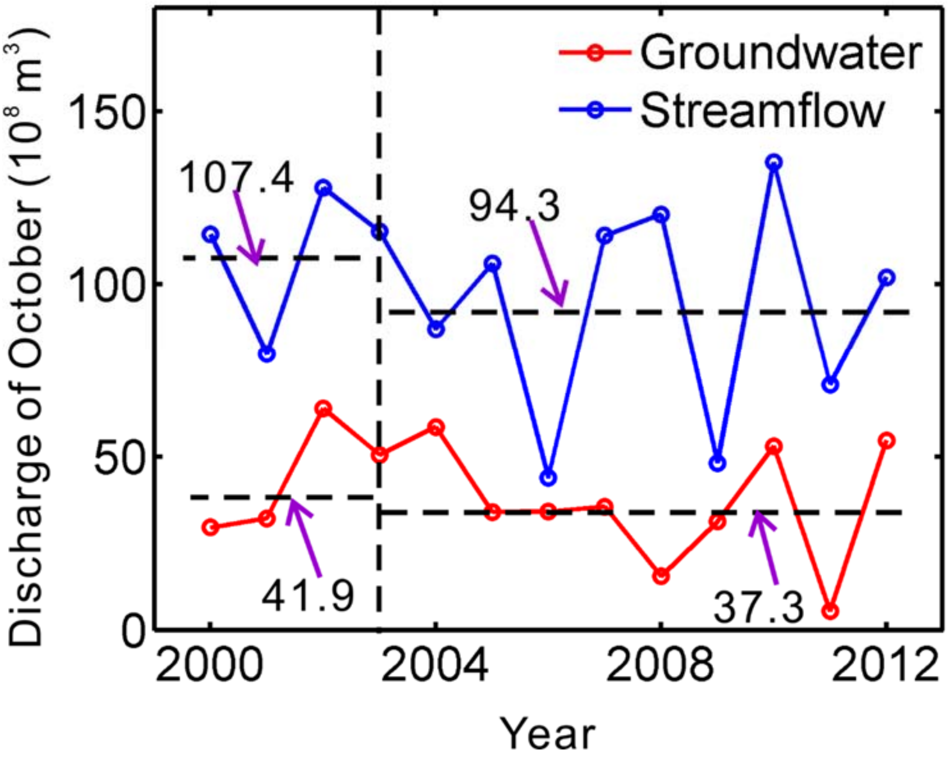

Fig. S14. Monthly streamflow and groundwater in October at Hukou station. The figure was created with Matlab R2009b.

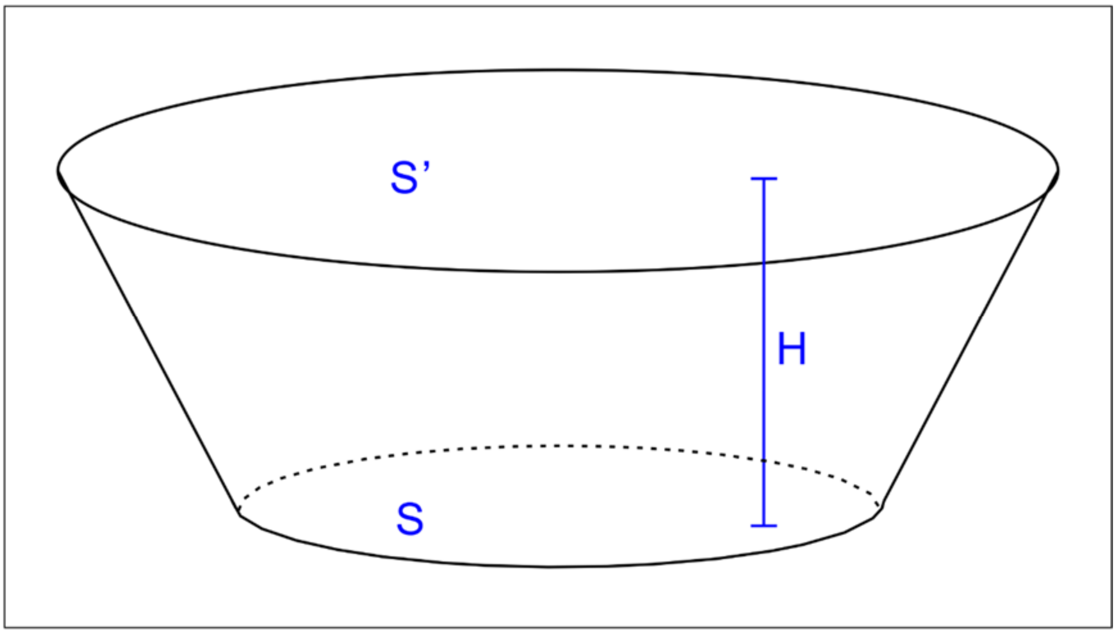

Fig. S15. Sketch of a frustum, which is used to calculate the water level with respect to a specific water volume. The figure was created with CorelDRAW X5.

Supplementary References

[1] Sen, P.K. Estimates of the regression coefficient based on Kendall's tau. *J Am Stat Assoc* **63,** 1379–1389 (1968).

[2] Tabari, H. & Talaee, P. H. Temporal variability of precipitation over Iran: 1966–2005. *J Hydrol* **396,** 313–320 (2011).

[3] Alexandersson, H. & Moberg, A. Homogenization of Swedish temperature data, Part I: Homogeneity test for linear trends. *Int J Climatol* **17,** 25-34 (1997).

[4] IPCC. Climate Change. The IPCC Third Assessment Report. Volumes I (Science), II (Impacts and Adaptation) and III (Mitigation Strategies). Cambridge Univ. Press, Cambridge. (2001).

[5] de Leeuw, J. et al. Strategic assessment of the magnitude and impacts of sand mining in Poyang Lake, China. *Reg Environ Change* **10,** 95-102 (2010).

[6] Xu L. et al. Analysis of water balance in Poyang Lake Basin and subsequent response to climate change, *J Coastal Res* **68 (sp1),** 136-143 (2014).

[7] Jiangxi water resources bulletin 2011. Press of Water Resources Department of Jiangxi Province.

[8] CSRWRB (Changjiang and Southwest Rivers water Resources Bulletin), 2008–2010. Wuhan: Changjiang Press.

[9] Dai, Z., Liu, J.T. and Xiang, Y.B. Human interference in the water discharge of the Changjiang (Yangtze River), China. *Hydrolog Sci J* (2014)

[10] Min Q., Su Z. and Wang X. Characteristics and causes of Poyang Lake surface evaporation variation in recent 50 years. *Meteorology and disaster reduction research* **30(3),** 17-20 (2007). (in Chinese with English abstract)

[11] Ye X. et al. Variation of reference evapotranspiration and its contributing climatic factors in the Poyang Lake catchment, China. *Hydrol. Process.* **28,** 6151-6162 (2014).

[12] Dai, Z. et al. Assessment of extreme drought and human interference on baseflow of the Yangtze River. *Hydrol. Process* **24,** 749-757 (2010).

[13] Arnold, J.G. et al. Regional estimation of base flow and groundwater recharge in the Upper Mississippi river basin. *J Hydro.* **227,** 21-40 (2000).

[14] Nathan R.J.& McMahon, T.A. Evaluation of automated techniques for base flow and recession analysis. *Water Resour Res* **26 (7),** 1465–1473 (1990).

[15] Tan, G.L. Research on the evolution of hydrology and water resources ecological economic zone of Poyang Lake. Beijing: China Water Conservancy and Hydropower Press 130-133 (2013). (in Chinese)
